# Prognostic significance of STAT3 gene expression in patients with glioblastoma tumors: a study from Western India

**DOI:** 10.1186/s43046-022-00133-4

**Published:** 2022-07-18

**Authors:** Trupti Trivedi, Kinjal Panchal, Neha Bhalala, Priti Trivedi

**Affiliations:** 1 Molecular Diagnostics & Research Laboratory I, The Gujarat Cancer & Research Institute, Ahmedabad, Gujarat India; 2 Molecular Diagnostics & Research Lab-I, Cancer Biology Department, Gujarat Cancer & Research Institute, Asarwa, Ahmedabad, Gujarat 380 016 India; 3 Department of Oncopathology, The Gujarat Cancer & Research Institute, Ahmedabad, Gujarat India

**Keywords:** STAT3, IDH, Glioblastoma, Multivariate, Prognostic significance, Tumor Location

## Abstract

**Abstract:**

**Objective:**

Glioblastoma Multiforme (GBM), a devastating the most common primary malignant intracranial brain tumors. In India, the incidence of this malignancy is escalating, however, there are very few studies on this tumor entity from Indian population. The present study sought to investigate the prevalence and prognostic significance of Signal Transducer and Activator of Transcription 3 (STAT3) gene expression in GBM patients from Western India.

**Method:**

STAT3 gene expression using real-time PCR was detected in total 55 GBM patients. The impact of STAT3 aberrant expression on progression-free survival (PFS) and overall (OS) was analysed using univariate and multivariate survival analysis. The data were analysed using SPSS statistical software and p value ≤0.05 was considered as significant.

**Results:**

The aberrant STAT3 expression was found in 85% (47/55) of patients with -1.12 fold change down-regulation in 49% (23/47) and 3.36 fold change up-regulation was noted in 51% (24/47) of patients. In wild type IDH tumors (*n*=30), down regulation and up regulation of STAT3 was noted in 63% and 27% of patients, respectively, whereas, for IDH mutant GBM tumors (*n*=25), the incidence of low expression and high expression of STAT3 was noted in 16% and 68% of patients, respectively. Thus, we found that incidence of STAT3 down regulation was significantly high in patients with IDH wild type tumors, whereas, in IDH mutant GBM tumors, the incidence of up-regulated STAT3 was significantly high (P=0.021, χ2=12.81, r=+0.310). In Kaplan-Meier univariate survival analysis, a part from age (P=0.006), tumor location (*P*=0.025), and KPS score (P=0.002), co-detection of STAT3 up regulation and presence of IDH mutation (*P*=0.030) remained significant prognostic factors for PFS and OS. In multivariate survival analysis also, co-detection of STAT3 high expression and presence of IDH mutation remained independent prognosticators for PFS (HR=6.45, 95% CI=1.32-31.40, *P*=0.021) and OS (HR=8.69, 95% CI=1.66-45.51, *P*=0.010).

**Conclusion:**

For GBM tumors, STAT3 up-regulation and presence of IDH mutations together predicts better survival. This reflects unique molecular etiology for GBM patients. Therefore, they would be useful in the future for targeted therapy and for clinicians they would be useful for better patient management. However, study on a larger sample size is required for validation.

## Background

Glioblastoma Multiforme (GBM) tumors are the most common primary malignant intracranial tumors of the brain, characterized by extremely aggressive biological behaviour, early recurrence and often break prognosis [1]. Even, individual tumors with a specific histopathological entity, may behave differently clinically, and have variable response to therapy [2]. Currently, for GBM tumors, IDH (Isocitrate Dehydrogenase) is the only driver gene that helpful to classify GBM patients into primary and secondary GBM, however, the genetic basis and molecular pathways for the development of primary and secondary GBM are different [3]. Thus, these tumors have tremendous inter-tumoral molecular heterogeneity, therefore, despite of current treatment modalities in various combinations, there has been only a marginal improvement in survival [1, 2]. This indicates that currently for GBM patients, there are no curative treatment options available. Thus, not only an appropriate therapy of these patients still remains a major challenge, but also novel drug targets for personalized therapy are also eagerly awaited and remained a major challenged [4].

Worldwide, this devastating tumor entity represents a major threat to the public health system due to its high level of morbidity and mortality [5]. Importantly, the incidence of this malignancy is escalating in developing countries, including, India. A recently published report in Lancet by Patel et al (2019) has shown that India is in the top three countries where the incident cases of Central Nervous System (CNS) tumors in both sexes have been reported [6]. In India, the incidence of these tumors is 1–4/100,000 cases [7]. However, since India does not have a centralized cancer registration system, population based cancer registries representing a small population is the only option. This makes epidemiological studies incomplete and hard to even perform, data may provide only a skewed understanding of incidence and mortality [7]. Our institute is a Regional Cancer Centre of Western India, and here the cases of brain tumors registered were 1.81% of all malignancies. GBM accounts for 18% of all primary brain tumors and 45.9% of all glioma tumors (Data from Population-based Cancer Registry, GCRI). Thus, overall, the epidemiological data of GBM tumors indicates that the incidence of this malignancy is increasing in India. However, there are very few studies on this tumor entity from Indian population [8] which is really worrisome for GBM patients and for clinicians also. Therefore, it is extremely important to identify the potential molecule for GBM tumor entity that could be useful to reduce the morbidity and mortality of patients and could be useful in future as a targeted therapy for better patient management.

STAT3 (Signal Transducer and Activator of Transcription 3) is a member of the STAT family, and transcription factor. Among the STAT proteins, STAT3 has received the most scrutiny because of its pleiotropic functions in diverse biological settings. Within the nervous system, STAT3 signaling plays an instructive role in astrocyte differentiation and has been found to play dual roles as tumor-suppressive and oncogenic in glial malignancy, most importantly depending on the mutational profile of the tumor [9]. Thus, in a multistep way, the activated STAT3 has a role in initiating and promoting gliomagenesis**.** However, concerning its association with the clinical outcome of GBM patients, there is a paucity of data has been reported. Therefore, the current study was aimed to explore the prevalence of STAT3 gene expression in GBM tumors and to determine their impact on the prognosis of GBM patients from Western India. To the best of our knowledge, no such study has been reported from Western India in the past. Therefore, this is the first time data is being reported for GBM patients from the West Zone.

## Methods

### Patient selection and follow-up details

A total of 55 untreated newly diagnosed histologically confirmed astrocytoma grade 4 (GBM) patients were enrolled and included from January 2017 to January 2020 in the current study. The study was approved by the Institutional Review Board and Ethics Committee and written consent forms were obtained from all the patients prior to treatment administration. Formalin-fixed and paraffin-embedded tissue blocks of GBM tumors and normal tissue samples were obtained from the histopathology department of the Institute. Detailed clinical and pathological history of the patients was obtained from the case files maintained at the Medical Record Department of our institute. The clinical-characteristics of the enrolled patients are enlisted in Table 1.

**Table 1:** Clinical characteristics of GBM patients

Characteristics	N	%
Total Patients	55	
Age (Median: 50 years, range: 24-70 years)		
≤50	27	49
>50	28	51
Gender		
Female	20	36
Male	35	64
Tumor Location		
Frontal	23	42
Temporal	12	22
Parietal	17	31
Occipital	03	05
KPS^a^ Score (Median: 60)		
High KPS:>60	31	56
Low KPS:<60	24	44
Treatment		
Only Surgery	34	62
Followed by		
Radiotherapy (RT)	04	07
Chemotherapy (CT)	06	11
RT + CT	11	20
Progression-free survival (*n*=34)		
Median 12 months		
GC well	24	71
Recurrence	10	29
Median 24 months		
GC well	14	41
Recurrence	20	59
Overall survival (*n*=34)		
Median-12 months		
Alive	27	79
Died	07	21
Median-24 months		
Alive	11	32
Died	23	68
IDH1/2 Mutations		
Absent	30	55
Present	25	45
STAT3 gene expression		
Normal Expression	08	15
Altered expression	47	85
Down regulation	23	49
Up-regulation	24	51

^a^Karnofsky Performance Status

Survival analysis for progression-free survival (PFS) and overall survival (OS) was evaluated for 12 months and 24 months in total 34 patients who could be followed for a minimum period of 24 months or until their death within that period. Within 12 and 24 months, 29% and 59% of patients had developed recurrence, respectively.

Death incidence was reported in 21% and 68% within 12 and 24 months, respectively Table 1.

DNA and RNA were extracted from FFPE blocks of the same patients, using QIAmp DNA FFPE 86 Tissue Kit (Qiagen, Germany) and the Norgen Biotech FFPE RNA purification kit (Norgen, Canada), respectively. DNA extraction was done for IDH1/2 mutation detection and RNA extraction was done to analyse the STAT3 gene expression using real time PCR. The concentration of DNA and RNA was measured with Qubit Fluorometer (Invitrogen, Carlsbad, CA, USA) and the integrity of DNA was evaluated through agarose gel electrophoresis.

### ARMS Real-time PCR for IDH1/2 mutation detection

IDH1/2 mutations was detected using ARMS PCR using IDH1/2 RGQ PCR kit following manufacturer’s instructions (Qiagen). Qualitative detection of 6 mutations within IDH1 codon 132, one within IDH1 codon 100 (R100Q) and 5 within IDH2 codon 172 was noted. PCR was performed using the Rotor-Gene Q 5-plex HRM instrument (Qiagen). The PCR condition used was: 95°C Time: 10 min Cycling 40 times 95°C for 15 sec 60°C for 60 sec with an acquisition of FAM™ fluorescence in channel Green: Single. Sample ΔCt values were calculated as the difference between the mutation assay Ct and respective total assay Ct from the same sample. Samples were classified as mutation positive if the ΔCt value was less than or equal to the ΔCt cut-off value of the respective mutation assay [10].

### STAT3 gene expression using real time PCR

Total RNA (1μg) was reverse transcribed to generate complementary DNA (cDNA) using the Quanti Nova cDNA Reverse Transcription Kit (Qiagen) according to the manufacturers’ instructions. For relative expression of STAT3 gene, qPCR was performed in 20 μl reaction volumes using SYBR green master mix (Qiagen, USA) kit on Rotor-Gene Q Real-Time PCR instrument (Qiagen, Germany). Each reaction contained 2μl cDNA, 10μl SYBR Green PCR Master Mix, 0.4 μM forward and reverse primers. Primer sequences are in Table 2.

**Table 2:** Primer sequences of Stat3 and housekeeping genes

Name of genes	Forward sequence	Reverse sequence
STAT3	AGCAGCTTGACACACGGTA	GCCCAATCTTGACTCTCAATCC
18 S RNA	GGAGTATGGTTGCAAAGCTGA	ATCTGTCAATCCTGTCCGTGT

The reactions were carried out at 95°C for 2 min, followed by 45 cycles of denaturation at 95°C for 5s, annealing at 60°C for 10 sec and extension at 95°C for 30 sec. Relative gene expression of STAT3 was calculated by the 2-ΔΔCt method by using 18s RNA as a house-keeping gene. The reaction was performed in triplicates. The threshold for cycle of threshold (Ct) analysis of all samples was set at 0.05 relative fluorescence units in Rotor Gene Q system. The Ct value of the target gene STAT3 was normalized with the Ct value of 18s RNA. The mean of Ct values of STAT3 gene and mean of Ct values of 18s RNA gene were compared.

The calculation was based on ΔΔCt or Livak method [11]. The formulas of the Livak method were added in the excel sheet and fold change values of all tumor samples were calculated as compared to the reference gene and also as compared to normal brain tissue. The results were compared with normal brain tissue samples. One fold change was obtained for normal samples, fold change above 1 showed the up-regulation and the fold change value below 1 was considered down-regulation [12].

### Statistical analysis

Statistical analysis was carried out using SPSS (Statistical Package for the Social Sciences) statistical software version 21.0. Paired *t*-test was used to compare the mRNA levels of STAT3 from GBM tumors and normal brain tissues. Non-parametric Mann-Whitney ‘U’ test was performed to evaluate the significance of STAT3 gene expression between the groups. Two-tailed χ2 test was used to assess the association between two parameters. Correlation between two parameters was calculated using Spearman’s correlation coefficient (r). Kaplan-Meier survival analysis was performed for overall survival (OS). Multivariate survival analysis was performed using Cox Proportional Stepwise Hazard Regression Model. The Wald statistic and Hazard Ratio (HR) with 95% confidence interval (CI) were used to assess risk for overall survival. P values ≤0.05 were considered significant.

## Results

### Prevalence of STAT3 gene expression based on IDH mutational status of GBM

**Fig 1:**
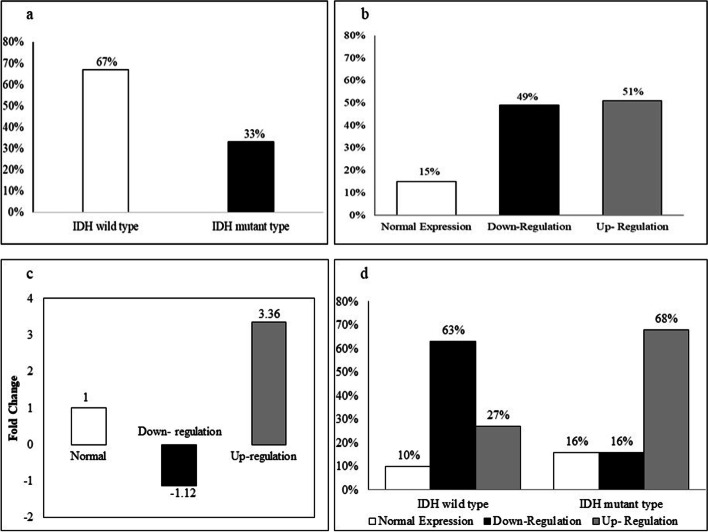
Prevalence of STAT3 gene expression in GBM tumors. **a** IDH mutational status in GBM tumors, **b** Prevalence of STAT3 gene expression in GBM tumors, **c** Fold change expression of STAT3 gene in GBM, **d** STAT3 gene expression based on IDH mutational status

**Fig 2:**
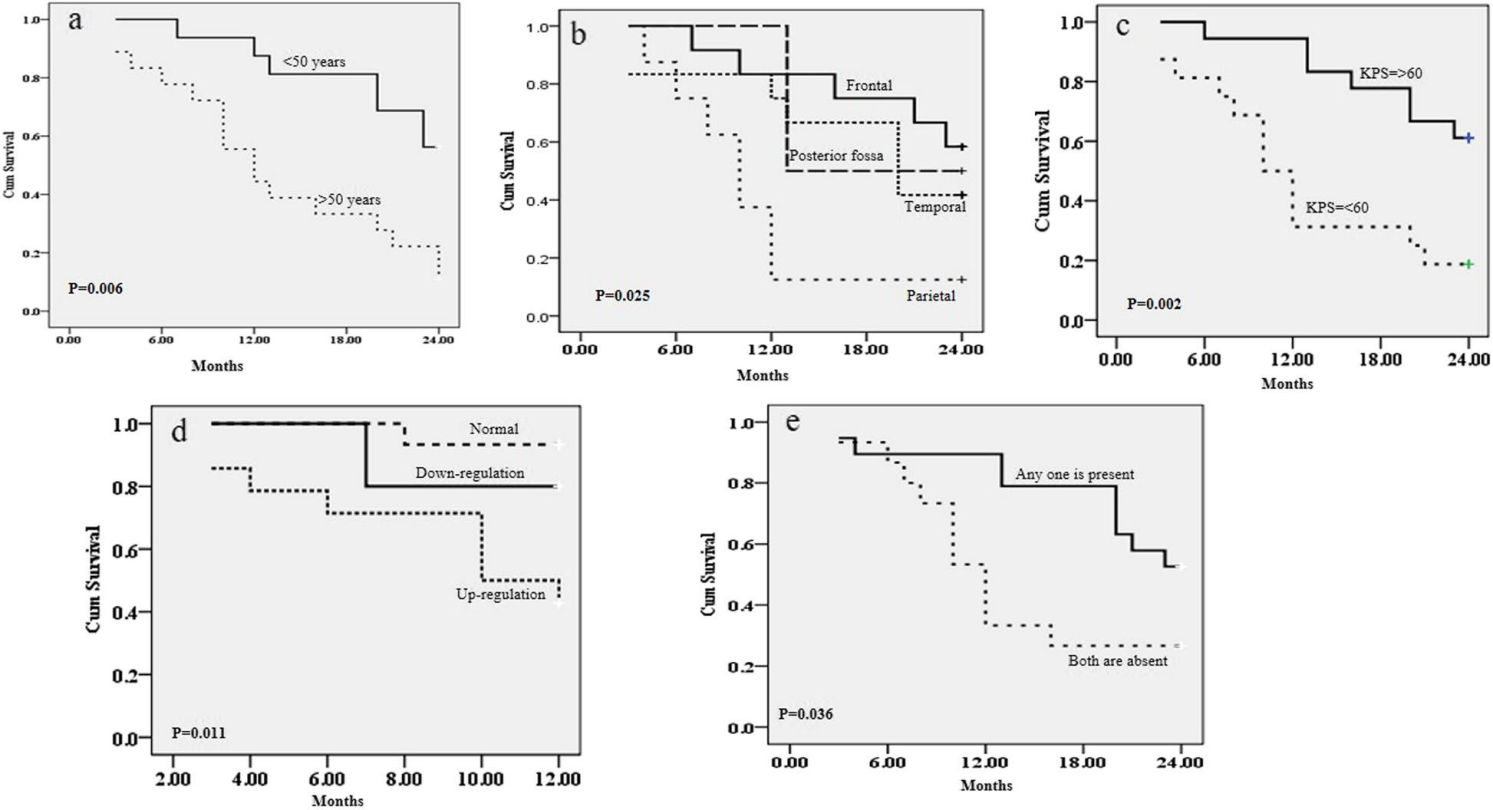
Kaplan-Meier Survival curves for Progression-free survival. **a** Patients with >50 years age had significantly reduced PFS. **b** Patients whose tumors from temporal site showed high incidence of relapsed than patients with GBM tumors from parietal, occipital and frontal. **c** Patients with <60 KPS, had significantly high incidence of recurrence within 24 months. **d** Patients with presence of up-regulated STAT3 had better 12 months PFS. **e** Co-detection of STAT3 up-regulation and IDH mutation showed better 24 months PFS

Fold change and relative expression of STAT3 gene in GBM tumors were carried out (Fig. 1b and c). Out of 55 patients, 15% (8/55) of patients showed normal expression STAT3 gene, whereas as compared to normal, the aberrant gene expression of STAT3 was found in 47 patients. Thus, 85% of patients had either down-regulation or up-regulation of STAT3 gene expression. In these patients, -1.12 fold change down-regulation of STAT3 gene was observed in 49% (23/47) and 3.36 fold change up-regulation was noted in 51% (24/47) of patients (Fig. 2c). Then we categorized STAT3 gene expression based on IDH mutational status in GBM. In patients with wild type IDH tumors (*n*=30), normal expression of STAT3 gene was observed in 10%, (3/30) patients. However, down regulation (low expression) and up regulation (high expression) of STAT3 was noted in 63% (19/30) and 27% (8/30) of patients, respectively. For patients with IDH mutant GBM tumors (*n*=25), the incidence of low and high expression of STAT3 was noted in 16% (4/25) and 68% (17/25) of patients, respectively (Fig. 1d). Interestingly, we found statistically significant difference in incidence of STAT3 gene expression between IDH wild type and mutant type GBM tumors. The incidence of STAT3 down regulation was significantly high in patients with IDH wild type tumors, whereas, in IDH mutant GBM tumors, the incidence of up-regulated STAT3 was significantly high (*P*=0.021, χ2=12.81, r=+0.310) Table 3. Thus, we observed linier correlation between STAT3 up-regulation with IDH mutation.

**Table 3:** Inter-correlation between STAT3 gene expression and IDH mutation in GBM tumors

	N	IDH mutation status	
	55	Wild type ***N***=30	Mutant type ***N***=25
**STAT3 gene expression**	**N (%)**	**N (%)**	**N (%)**
Normal expression	07 (13)	03 (10)	04 (16)
Down regulation	23 (42)	19 (63)	04 (16)
Up-regulation	25 (45)	08 (27)	17 (68)
		**P=0.021** **χ**^**2**^**=**12.81, r=+0.310	

### Correlation between STAT3 gene expressions with clinical characteristics of GBM tumors

No significant correlation was noted between altered expression of STAT3 gene with any of the clinical characteristics, such as age, gender, location of tumors, and KPS scale.

### Univariate survival analysis for PFS and OS using Kaplan-Meier Survival Analysis

Univariate survival analysis for PFS demonstrated that patients with age >50 years (12 months: log rank=4.28, *P*=0.038, 24 months: log-rank=7.52, *P*=0.006), low KPS (12 months: log-rank=6.35, *P*=0.012, 24 months: log-rank=9.57, *P*=0.002), absence of IDH1/2 mutations (12 months: log-rank=9.67, *P*=0.002, 24 months: log-rank=7.25, *P*=0.007), down regulated (low expression) STAT3, (12 months: log-rank=9.04 *P*=0.011), and co-detection of low expression of STAT3 and absence of IDH 1/2 mutations (12 months: log-rank=6.44, *P*=0.011, 24 months: log-rank=4.73, *P*=0.030) were found having high incidence of relapsed within 12 months and 24 months (Fig. 2a). Moreover, location of tumors showed significant difference in the incidence of progression of disease within 24 months. Accordingly, high incidence of relapsed was noted in patients with temporal tumors followed by parietal, occipital and frontal tumors (log rank=9.38, *P*=0.025) However, it was failed to predict reduced 12 months PFS (Table 4, Fig 2a to e).

**Table 4:** Univariate survival analysis for PFS and OS using Kaplan-Meier Analysis (*n*=34)

		Progression-free survival				Overall survival			
		12 months		24 months		12 months		24 months	
Parameters		Disease relapsed	Log rank ***P*** value	Disease relapsed	Log rank ***P*** value	Patients died	Log rank ***P*** value	Patients died	Log rank ***P*** value
	N	N (%)		N (%)					
**Age**									
≤50	16	02 (13)	**4.28** **0.038**	06 (38)	**7.52** **0.006**	01 (06)	**3.72** **0.050**	07 (44)	**9.51** **0.002**
>50	18	08 (45)		14 (78)		06 (33)		16 (89)	
**Tumor Locations**									
Frontal	12	03 (25)	NS^a^	05 (42)	**9.38** **0.025**	02 (17)	NS	06 (50)	**11.92** **0.008**
Temporal	08	05 (63)		07 (88)		03 (38)		08 (100)	
Parietal	12	02 (17)		07 (58)		02 (17)		08 (67)	
Occipital	02	00 (00)		01 (50)		00 (00)		01 (50)	
**KPS**^**b**^ **Scale**									
High >60	18	02 (11)	**6.35** **0.012**	07 (39)	**9.57** **0.002**	01 (06)	**5.13** **0.024**	10 (56)	**6.88** **0.009**
Low <60	16	08 (50)		13 (81)		06 (38)		13 (81)	
**IDH½ mutation status**									
Absent	19	10 (53)	**10.63** **0.001**	13 (68)	**4.58** **0.032**	07 (37)	**8.11** **0.017**	14 (74)	NS
Present	15	00 (00)		07 (47)		00 (00)		09 (60)	
**STAT3 gene expression**									
Normal expression	05	01 (20)	**9.042** **0.011**	04 (80)	NS	01 (20)	NS	04 (80)	**4.39** **0.036**
Down regulation	14	08 (57)		09 (64)		06 (43)		10 (71)	
Up regulation	15	01 (07)		07 (47)		00 (00)		09 (60)	
**High STAT3 & Mutant IDH together**									
Both are absent	15	08 (53)	**6.44** **0.011**	11 (73)	**4.73** **0.030**	05 (33)	**9.67**	12 (80)	**6.38** **0.005**
Any one is present	19	02 (11)		09 (47)		02 (11)	**0.002**	11 (58)	

^a^*NS* Not Significant, ^b^*KPS* Karnofsky Performance Status

For OS, univariate survival analysis indicated that patients with age >50 years (12 months: log-rank=3.72, *P*=0.050, 24 months: log-rank=9.51, *P*=0.002), location of tumors (24 months; log rank=11.92, *P*=0.008), low KPS (12 months: log-rank=5.13, *P*=0.024, 24 months: log-rank=6.88, *P*=0.009), low expression of STAT3 gene (24 months: log-rank=4.39, *P*=0.036), absence of IDH1/2 mutations (12 months: log-rank=8.11, *P*=0.017) and co-detection of low STAT3 gene expression and absence of IDH1/2 mutations (12 months: Log-rank=9.67, P=0.002, 24 months: log-rank=6.38, P=0.005) showed high incidence of death as compared to their respective counterparts, and was found to be associated significantly with better OS.

**Fig 3:**
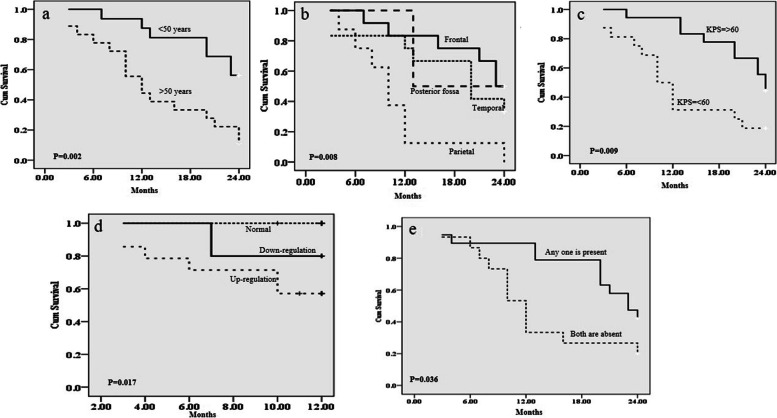
Kaplan-Meier Survival curves for overall survival. **a** Patients with >50 years age had significantly inferior OS. **b** Patients whose tumors from temporal lobe had significantly shorter OS as compared to patients with parietal, occipital and frontal lobes. **c** Patients with <60 KPS, had significantly high incidence of death within 24 months. **d** Within 12 months, low incidence of death was noted in patients with presence of up-regulated STAT3. **e** Significantly low death rate was observed in patients with presence of co-detection of STAT3 up-regulation and IDH mutation

However, expression of STAT3 and IDH mutation failed to predict 24 months OS, where the location of tumors showed a significant difference in the incidence of death within 24 months (Table 4, Fig 3a to e).

### Multivariate survival analysis using Cox-proportional Step-wise Hazard Model for PFS and OS

Multivariate survival analysis for PFS demonstrated that KPS (HR=4.20, Wald=8.51, 95% CI=1.60-11.03, *P*=0.004) entered in the equation at step 1 followed by age (HR=3.78, Wald statistics=6.90, 95% CI= 1.40-10.23, *P*=0.009) at step 2 for predicting reduced 24 months PFS. Additionally, combined detection of STAT3 gene up regulation and presence of IDH gene mutation showed significant association with better 24 months PFS after adjusting KPS score and age (HR=6.45, Wald=5.32 95% CI=1.32-31.40, P=0.021) Table 5. For OS, similar results we observed with STAT3 high expression and IDH mutation remained significantly positive prognosticators at step 1 for both 12 months and 24 months. The KPS score emerged significant marker at step 2 for predicting 24 months shorter OS (Table 5).

**Table 5:** Multivariate survival analysis for PFS and OS using Cox Forward Stepwise Hazard Proportional Model for GBM patients

Survival	Step	Parameter	HR	Wald	Lower	Upper	***P*** value
**PFS**							
Months							
24	1	KPS^b^ Scale	4.20	8.51	1.60	11.03	**0.004**
	2	Age	3.78	6.90	1.40	10.23	**0.009**
		High STAT3+Mutant IDH^a^	6.45	5.32	1.32	31.40	**0.021**
**OS**							
Months							
12	1	High STAT3+Mutant IDH	8.69	6.55	1.66	45.51	**0.010**
24	1	High STAT3+Mutant IDH	3.64	7.93	1.48	8.96	**0.005**
	2	KPS	3.42	7.32	1.40	8.36	**0.007**

^a^After adjusting for KPS and Age, ^b^*KPS* Karnofsky Performance Status

## Discussion

Although, remarkable improvement has been made over the last decades in the understanding of the pathogenesis of GBM tumors, the patients with these tumors still die within a shorter period of time, even after first line treatment. Although multiple studies have been conducted concerning the prognostic importance of different variables over the decades, the picture remains incomplete and unclear.

In the current study, we analyzed STAT3 gene alterations in astrocytic grade 4 glioma tumors. The altered expression of STAT3 gene was noted in 85% of GBM tumors. We observed STAT3 gene expressions between IDH mutant and IDH wild-type tumors. We found statistically significant difference in altered STAT3 gene expression incidence between IDH mutant and wild type GBM tumors. Thus, our results demonstrated that though, histopathologically astrocytoma grade 4 GBM tumors are a single entity, their molecular mechanisms distinctly differ based on IDH mutational status. Interestingly, we found positive correlation between up-regulated STAT3 with mutated IDH. Also, multivariate survival analysis for PFS and OS shown that combined detection of upregulated STAT3 gene expression and presence of IDH mutation were significant positive prognosticators for predicting longer 12 months and 24 months PFS and OS. Thus, this preliminary study indicated that along with IDH mutational status, study of STAT3 gene expression might be useful to identify subgroup of GBM patients for better treatment management and in the future STAT3 might be useful for targeted therapy. However, validation is required in more number of GBM patients.

STAT3, a family of transcription factors, plays a central role in neural stem cell and astrocyte development [9]. Recent studies have uncovered that STAT3 functions are multifaceted and not easy to classify. The specific cellular role of STAT3 seems to be determined by the integration of multiple signals, by the oncogenic environment, and by the alternative splicing into two distinct isoforms, STAT3α and STAT3β. Based on these different conditions, and the genetic background of the tumor STAT3 can act both as a potent tumor promoter or tumor suppressor factor. However, in GBM very few study reports of STAT3 are noted [13]. In the present study, aberrant STAT3 expression was observed in 85% of GBM tumors. Considering one fold change for normal brain tissue samples, the fold change below 1 was considered as down regulation (low expression) of STAT3 and the fold change value above 1 was considered up-regulation of this gene [12]. Accordingly, the down regulation (-1.12) of STAT3 was noted in 49% of tumors and up regulation (3.36) of STAT3 was noted in 51% of GBM tumors. The possible reason for down regulation of STAT3 in wild type GBM, is inactivation of PTEN (Phosphatase and Tensin Homolog) mutation, consequent AKT (Protein Kinase B) activation leading to down-regulation of cytokine receptor leukemia inhibitory factor receptor and inhibition of STAT3 signaling, thereby leading to IL-8 (Interleukin 8) induced proliferation and invasiveness [14]. There was an interesting finding we noted when we categorized down and up-regulated status of STAT3 with IDH mutational status. We found linear correlation between up-regulated STAT3 and the presence of mutant IDH (P=0.021). This is indicates that in IDH-mutant GBM tumors, the STAT3 signaling pathway remains activated with high STAT3 gene expression. It has been observed that IDH mutated gliomas are slow-growing brain tumors, which further progress into high-grade glioma. Recently, Leventoux et al (2020) have reported that 20% of tumors with the presence of IDH mutation already have transformation foci. In their work, they have shown that STAT3 signaling pathway promotes IDH mutant glioma tumors to further progress into high-grade gliomas. Thus, in concordance to our findings their study also demonstrated that in IDH mutant GBM, STAT3 signaling pathway remains activated [15].

From the prognostic perspective, the univariate Kaplan-Meier survival curves for PFS demonstrated that apart from age, KPS, location of tumors, presence of IDH mutation, the up-regulation of STAT3 gene and combination of up regulated STAT3 with presence of IDH mutation have remained significant prognostic parameters that identify subgroup of GBM patients with longer PFS and OS. Moreover, for GBM patients, age and KPS are the most common prognostic parameters. Interestingly, we found tumor location was a significant prognosticator for GBM patients. In our previous studies on oral cancer and glioma, we have reported that though histopathologically a single entity, locations of tumors has a significant impact on survival due to differences in biological behaviour [16, 10]. Similarly, in the current study, we observed significant differences in death incidence with various locations of GBM tumors. We noted that patients whose tumors were from frontal and posterior fossa of the brain showed significantly low incidence of death within 24 months in comparison to patients whose tumors were from parietal and temporal region (log rank=11.92, *P*=0.008). This is indicating towards tumor heterogeneity from various locations of the brain and different biological behaviours. Similar to this, recently, Fyllingen et al (2021) have shown a reduced OS in patients with tumors in the left temporal lobe compared to tumors in the dorso-medial right temporal lobe and the white matter region involving the left anterior paracentral gyrus/dorsal supplementary motor area/medial precentral gyrus [17]. Thus, overall, the location of GBM tumors has a significant impact on disease outcomes.

Up-regulated STAT3 also showed a significant association with longer PFS and OS in univariate survival analysis. Also, co-detection of high STAT3 and IDH mutation remained positive prognosticators for GBM patients in multivariate survival analysis. It is well known that IDH mutation is a good prognosticator for GBM, however, there is a paucity of data for STAT3 being reported as a prognostic marker in GBM. Many studies have uncovered that STAT3 expression during cortical development is associated with astrocyte differentiation providing one potential explanation for why activation of STAT3 in astrocytic gliomas might correlate with a better prognosis [18]. On the other hand, Wu et al (2016) have shown in their recent review article that overexpression of STAT3 had significantly shorter OS for various malignancies, including glioma, where STAT3 acts as an oncogene [19]. There are several possible explanations for the dual role of STAT3 in GBM. For instance, the wide variety of stimulating cytokines and growth factors or the versatile posttranslational modifications of STAT3. Another possible cause of this heterogeneity is its expression as different isoforms. Alternative splicing of this gene gives rise to two isoforms of STAT3, STAT3α and its truncated version STAT3β. Both isoforms are transcriptionally active and display distinct functions under physiological and pathological conditions. STAT3 is widely described as an oncogene whereas, STAT3β has gained attention as a potential tumor suppressor. STAT3β can also functionally compensate the loss of STAT3 during astrocyte differentiation, which is highly dependent on STAT3 activity [20, 21]. Thus, it might be assumed that in our study up-regulated STAT3 with better PFS and OS indicated that it might act as tumor suppressor for subgroup of GBM patients. However, data using variant forms of STAT3 gene is required. The limitation of our study was its small sample size, therefore, data further require to validate in more samples of GBM tumors.

## Conclusion

Based on our findings, we conclude that for GBM entity, there might be a correlation between up regulated STAT3 transcript and presence of IDH mutations. This reflects unique molecular etiology of GBM patients. Also, our data demonstrated that STAT3 up regulation and IDH mutations both together predicts better survival than only presence of IDH mutations. Thus, along with IDH mutation, STAT3 up regulation is a significant prognosticator for GBM patients and therefore, would be useful in the future for targeted therapy, which would be helpful to the clinicians for better patient care. However, study on a larger sample size is required for validation.

## Data Availability

The datasets generated and/or analysed during the current study are not publicly available but are available from the corresponding author on reasonable request.

## Abbreviations

STAT3: Signal Transducer and Activator of Transcription 3
GBM: Glioblastoma
IDH: Isocitrate Dehydrogenase
PCR: Polymerase Chain Reaction
PFS: Progression-Free Survival
OS: Overall Survival
HR: Hazard Ratio
CI: Confidence Interval
KPS: Karnofsky Performance Status
CNS: Central Nervous System
DNA: Deoxyribonucleic Acid
RNA: Ribonucleic Acid
FFPE: Formalin-Fixed Paraffin-Embedded
ARMS: Amplification-Refractory Mutation System
SPSS: Statistical Package for the Social Sciences
PTEN: Phosphatase and Tensin Homolog
AKT: Protein Kinase B
IL-8: Interleukin 8
